# Association between lactate-to-albumin ratio and 28-day ICU mortality in pediatric severe pneumonia patients

**DOI:** 10.1371/journal.pone.0331486

**Published:** 2025-09-12

**Authors:** Yang Zhang, Rubing Guo, Li Wang, Jihong Hu

**Affiliations:** 1 Scientific Research and Experimental Center, Gansu University of Chinese Medicine, Lanzhou, China; 2 Key Laboratory of Dunhuang Medicine, Ministry of Education, Gansu University of Chinese Medicine, Lanzhou, China; 3 Research Center of Traditional Chinese Medicine, Gansu University of Chinese Medicine, Lanzhou, China; 4 Clinical College of Chinese Medicine, Gansu University of Chinese Medicine, Lanzhou, China; 5 College of Public Health, Gansu University of Chinese Medicine, Lanzhou, China; 6 Laboratory and Simulation Training Center, Gansu University of Chinese Medicine, Lanzhou, China; Pelita Harapan University Faculty of Medicine: Universitas Pelita Harapan Fakultas Kedokteran, INDONESIA

## Abstract

**Introduction:**

The lactate-to-albumin ratio (LAR) has emerged as a promising prognostic biomarker in critical care, reflecting both metabolic dysfunction and inflammatory status. However, its prognostic value in pediatric severe pneumonia remains underexplored. This study aimed to investigate the association between LAR and 28-day ICU mortality in pediatric severe pneumonia patients using data from the Paediatric Intensive Care database.

**Methods:**

A retrospective cohort study was conducted involving 617 pediatric severe pneumonia patients admitted to the ICU between 2010 and 2018. LAR was calculated as the ratio of lactate (mmol/L) to albumin (g/dL). The primary outcome was 28-day ICU mortality. Cox proportional hazards regression models were used to assess the relationship between LAR and mortality, with restricted cubic splines (RCS) employed to explore potential nonlinear associations. Subgroup analyses were performed based on age, sex, and oxygenation status.

**Results:**

A significant linear relationship was observed between LAR and 28-day ICU mortality. Each unit increase in log (LAR) was associated with a 2.51-fold higher mortality risk (HR 2.51, 95% CI: 1.73, 3.65; P < 0.001). Kaplan-Meier analysis confirmed that patients in the highest LAR tertile had significantly lower survival probabilities compared to those in the lowest tertile (log-rank P < 0.001). Subgroup analyses revealed consistent associations across age, sex, and oxygenation status, with no significant interactions (all P for interaction>0.05).

**Conclusions:**

Higher LAR levels are independently associated with increased 28-day ICU mortality in pediatric severe pneumonia patients, demonstrating a linear relationship. These findings highlight LAR as a valuable prognostic tool for early risk stratification and clinical decision-making in this population. Further multicenter studies are needed to validate these results and explore interventions targeting LAR reduction to improve outcomes.

**Trial registration:**

Not applicable.

## 1 Introduction

Pediatric severe pneumonia remains a major global threat to children’s health, particularly in developing countries, where it is still a leading cause of child mortality. According to the World Health Organization (WHO), pneumonia accounts for 15% of deaths in children under five years of age [1,2]. In large countries like China, due to the large population base, pediatric severe pneumonia not only affects numerous families but also places a heavy burden on the healthcare system [3,4]. Timely and accurate prognostic assessment is crucial for guiding clinical treatment and resource allocation.

Lactate serves as a biomarker for tissue hypoxia and metabolic dysfunction, while albumin reflects nutritional status and inflammatory response [5,6]. The lactate-to-albumin ratio (LAR) has emerged as a novel prognostic biomarker, demonstrating unique value in critical care medicine [7–9]. LAR not only reflects an individual’s metabolic and inflammatory status but is also closely associated with disease severity and prognosis. For instance, in a study of critically ill coronary heart disease patients in the intensive care unit (ICU), elevated LAR predicted higher 28-day all-cause mortality, with its predictive value surpassing that of lactate levels alone [10]. Compared to traditional biomarkers such as C-reactive protein (CRP) and blood lactate levels, LAR provides more comprehensive prognostic information [11]. Additionally, LAR demonstrated excellent performance in predicting mortality in pediatric trauma patients, with an area under the curve (AUC) value of 0.958, significantly higher than other inflammatory markers [12]. These findings suggest that LAR may serve as a more integrated and reliable prognostic tool. In pediatric critically ill patients, the prognostic value of LAR is gradually being recognized. Studies have shown that LAR is closely associated with 28-day all-cause mortality in pediatric sepsis patients. In a study on pediatric septic shock, LAR achieved an AUC value of 0.91 for predicting 28-day all-cause mortality, demonstrating its superior prognostic predictive ability [13].

Although the prognostic value of LAR in adult critically ill patients has been extensively studied, its application in pediatric populations, particularly in pediatric severe pneumonia patients, remains underexplored. Based on this background, this study aims to investigate the relationship between the LAR and 28-day mortality in pediatric severe pneumonia patients.

## 2 Materials and methods

### 2.1 Data source

The data for this study were obtained from the Paediatric Intensive Care (PIC) database, a single-center database from the ICU of the Children’s Hospital affiliated with Zhejiang University School of Medicine [14]. It includes de-identified health-related data from over 10,000 pediatric patients admitted between 2010 and 2018. The PIC database contains information on demographics, vital signs, laboratory test results, symptoms, medication use, and mortality. All personal information was anonymized to protect patient privacy. Since all patient records in the PIC database were fully de-identified, the Ethics Committee of Zhejiang University School of Medicine deemed it unnecessary to obtain individual patient consent.

### 2.2 Study population, exposure, and outcome

**Fig 1:**
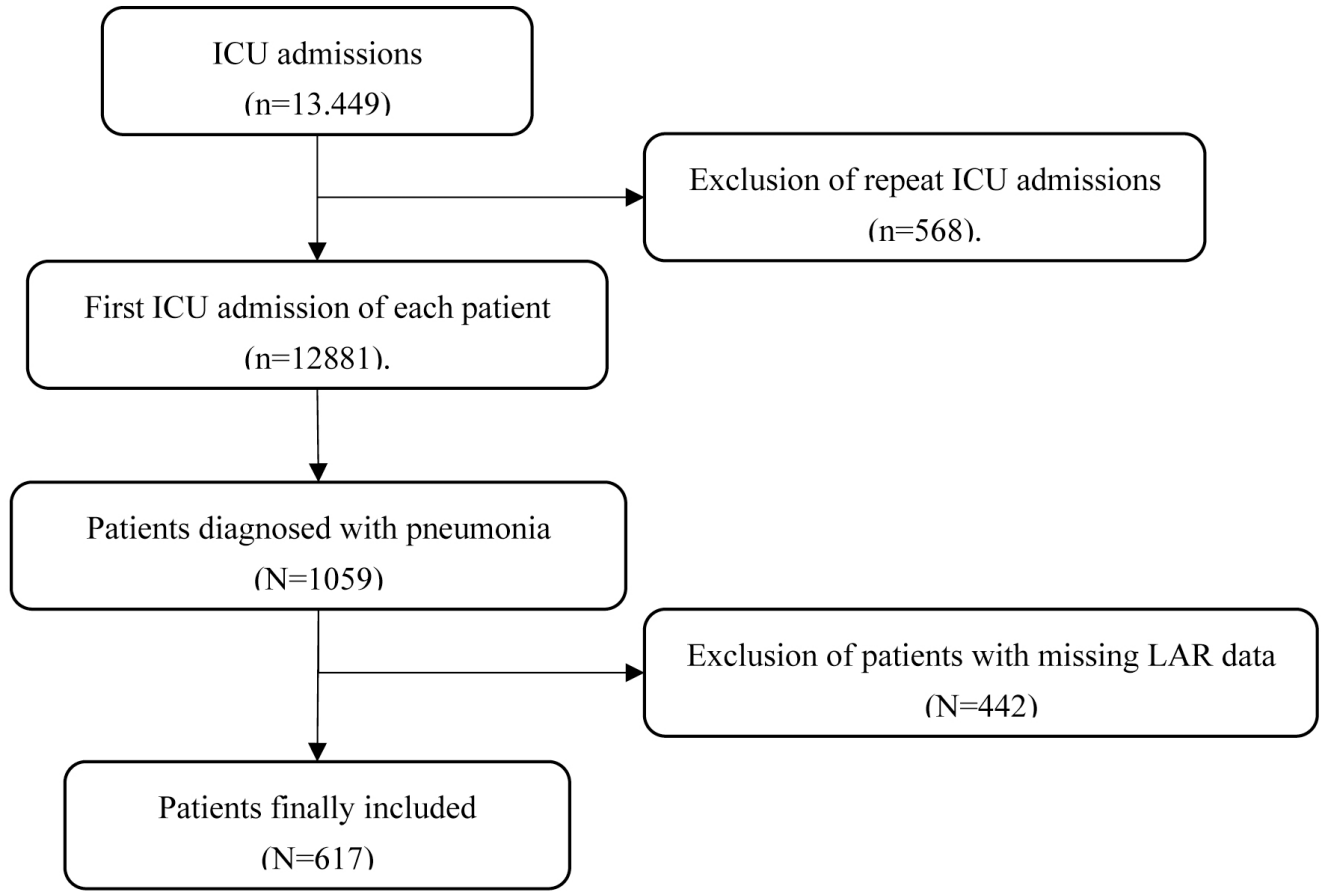
Study design and flowchart of study participants.

This study initially included 13,449 ICU admissions. After excluding 568 patients with repeated admissions, 12,881 patients with their first ICU admission were retained. Among the remaining patients, 1,059 were diagnosed with pneumonia based on ICD-10 codes. To ensure data completeness and accuracy, 442 patients with missing lactate or albumin data were further excluded. A total of 617 patients were ultimately included in the analysis (Fig 1). The LAR was calculated as the ratio of lactate (mmol/L) to albumin (g/dL), using the average lactate and albumin values measured within the first 24 hours of ICU admission. The primary outcome was 28-day ICU mortality, defined as the survival status within the ICU from the first day of admission to the 28th day of follow-up.

### 2.3 Data extraction

Relevant information was extracted from the PIC database using Navicat Premium software (version 17) by executing structured query language (SQL). Extracted data included age, sex, white blood cell count (WBC), red blood cell count (RBC), platelet count (PLT), hemoglobin, albumin, sodium, potassium, partial pressure of carbon dioxide (PaCO_2_), partial pressure of oxygen (PaO_2_), lactate, triglycerides (TG), total cholesterol, direct bilirubin, indirect bilirubin, alanine aminotransferase (ALT), aspartate aminotransferase (AST), and serum creatinine. All laboratory parameters were measured as the average value on the first day of ICU admission.

### 2.4 Statistical analysis

For continuous variables in baseline characteristics, the Kolmogorov-Smirnov test was first used to assess normality. Since all continuous variables were non-normally distributed, they were described as median (interquartile range), and group differences were compared using the Kruskal-Wallis test. Categorical variables were described as frequency (percentage), and group differences were compared using the chi-square test.

To evaluate the incidence of the primary outcome across different LAR tertiles, Kaplan-Meier survival analysis was performed, and the log-rank test was used to assess group differences. Due to the skewed distribution of LAR, natural logarithmic transformation (log (LAR)) was applied. The Cox proportional hazards model was used to estimate the hazard ratio (HR) and 95% confidence interval (CI) for the association between log (LAR) and the primary outcome. To prevent model overfitting, a formal variable selection procedure was implemented. We first assessed multicollinearity among potential covariates using the variance inflation factor (VIF). Subsequently, we employed the Least Absolute Shrinkage and Selection Operator (LASSO) Cox regression model with 10-fold cross-validation to identify the most influential predictors for inclusion in the final multivariate model. LAR was included as a categorical variable (with the lowest LAR tertile as the reference group). The p-value for trend was obtained by treating tertiles as an ordinal variable.

Additionally, restricted cubic splines (RCS) were used to explore potential nonlinear relationships between log (LAR) and 28-day mortality. The model was adjusted for all aforementioned covariates to ensure accuracy. To further assess the stability of this association, subgroup analyses were conducted based on age (<36 months vs. ≥ 36 months), sex, partial pressure of carbon dioxide (PaCO_2_: < 35 mmHg, 35–45 mmHg, > 45 mmHg), and partial pressure of oxygen (PaO_2_: < 90 mmHg, 90–100 mmHg, > 100 mmHg). Interaction p-values were calculated.

All statistical analyses were performed using R software (version 4.3.2, http://www.R-project.org), with statistical significance set at a two-sided p-value <0.05.

### 2.5 Ethical considerations

The research involving human participants was reviewed and approved by the Institutional Review Board of the Children’s Hospital, Zhejiang University School of Medicine (Hangzhou, China). In alignment with ethical guidelines for retrospective studies utilizing anonymized data from the PIC database, the ethics committee waived the requirement for informed consent, as the study did not impact clinical care or compromise participant privacy. This study adhered strictly to the ethical standards set forth in the Declaration of Helsinki (as revised in 2013).

## 3 Results

### 3.1 Baseline characteristics of individuals

This study involved 617 pediatric patients with severe pneumonia, with a 28-day ICU mortality rate of 9.40%. S1 Table compares baseline characteristics between survivors and non-survivors, showing significantly higher levels of LAR, lactate, ALT, AST, and PaCO₂ and lower PaO₂ in non-survivors (all p < 0.05). Table 1 presents baseline characteristics stratified by LAR tertiles, indicating that as LAR tertiles increased, WBC, direct bilirubin, indirect bilirubin, AST, and creatinine levels significantly increased, while median age, RBC, PLT, and PaO₂ significantly decreased (all p < 0.05).

**Table 1:** Baseline characteristics of participants.

Characteristic	LAR tertile			P-value
	Low (N = 206) (0.01–0.04)	Middle (N = 205) (0.04–0.06)	High (N = 206) (0.06–0.37)	
Age (months)	7.80 (2.55-23.40)	5.28 (2.04-21.24)	2.76 (1.32-11.37)	<0.001
Sex (Male)	119 (57.77%)	116 (56.59%)	129 (62.62%)	0.419
WBC (×10^9/L)	9.30 (6.68-13.36)	10.28 (6.64-14.16)	11.75 (7.71-16.95)	<0.001
RBC (×10^12/L)	3.91 (3.46-4.32)	3.77 (3.25-4.31)	3.60 (3.15-4.19)	0.019
PLT (×10^9/L)	314.50 (224.50-407.50)	311.50 (189.00-406.88)	245.75 (164.25-364.75)	0.004
HGB (g/L)	106.50 (97.25-119.00)	105.50 (91.62-119.94)	107.75 (92.00-125.88)	0.542
ALB (g/L)	38.50 (35.45-42.08)	37.10 (32.80-40.40)	35.05 (30.63-38.40)	<0.001
CHOL (mmol/L)	3.26 (2.70-4.11)	3.02 (2.38-3.61)	2.66 (1.84-3.29)	<0.001
TG (mmol/L)	0.88 (0.66-1.21)	0.86 (0.59-1.28)	1.01 (0.69-1.39)	0.031
BIL-DIR (μmol/L)	2.05 (1.28-4.17)	2.65 (1.60-6.20)	6.60 (2.30-13.20)	<0.001
BIL-INDIR (μmol/L)	4.82 (3.18-7.88)	5.10 (3.30-10.90)	9.40 (4.05-39.25)	<0.001
Scr (μmol/L)	38.00 (31.30-44.10)	40.00 (35.00-48.00)	46.75 (38.00-63.26)	<0.001
ALT (U/L)	21.00 (15.00-31.00)	20.00 (14.00-35.00)	22.00 (13.00-49.75)	0.419
AST (U/L)	40.00 (31.00-55.00)	46.00 (32.00-74.00)	59.50 (38.00-103.00)	<0.001
Na^+^ (mmol/L)	136.50 (134.54-138.79)	137.00 (135.00-139.40)	137.33 (135.00-139.81)	0.350
K^+^(mmol/L)	3.99 (3.70-4.32)	4.03 (3.70-4.33)	4.05 (3.61-4.50)	0.537
Lactate (mmol/L)	1.17 (1.00-1.35)	1.80 (1.60-2.00)	2.98 (2.45-3.84)	<0.001
PaCO_2_	44.91 (36.84-53.75)	42.70 (37.14-51.90)	42.05 (35.02-51.28)	0.149
PaO_2_	122.59 (90.54-158.92)	111.93 (74.30-142.67)	102.81 (73.12-133.20)	<0.001
Hospital days	12.17 (6.89-22.98)	11.90 (6.82-25.98)	12.94 (5.84-23.82)	0.407
ICU days	7.44 (3.67-16.39)	7.01 (3.86-15.88)	7.96 (3.01-17.00)	0.997
28-day ICU mortality	16 (7.77%)	23 (11.22%)	28 (13.59%)	0.161

Continuous variables in the table are expressed as median (interquartile range), while categorical variables are expressed as frequency (%). Abbreviations: LAR, lactate-to-albumin ratio; WBC, white blood cell count; RBC, red blood cell count; PLT, platelet count; HGB, hemoglobin; ALB, albumin; CHOL, cholesterol; TG, triglycerides; BIL-DIR, direct bilirubin; BIL-INDIR, indirect bilirubin; Scr, serum creatinine; ALT, alanine aminotransferase; AST, aspartate aminotransferase; Na^+^, sodium; K^+^, potassium; PaCO_2_, partial pressure of carbon dioxide; and PaO_2_, partial pressure of oxygen.

### 3.2 Relationship between LAR and 28-day ICU mortality

**Fig 2:**
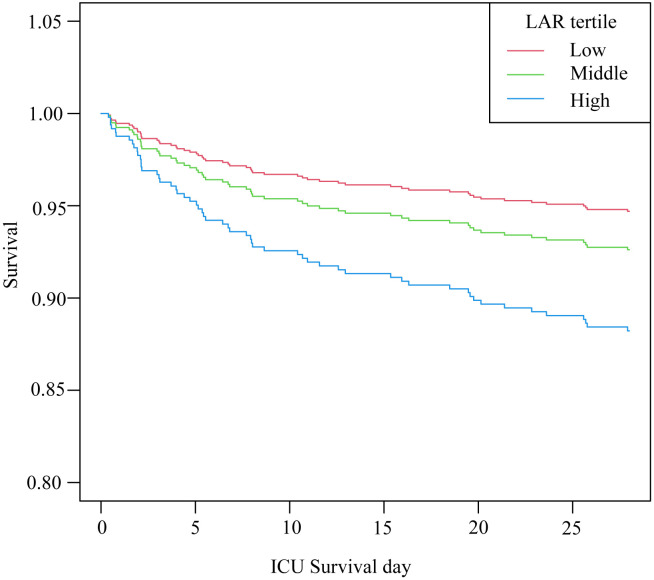
Kaplan-Meier analysis of 28-day survival rate stratified by tertiles of lactate-to-albumin ratio (log-rank test, p < 0.001).

Fig 2 shows the Kaplan-Meier survival curves stratified by LAR tertiles, revealing that the cumulative survival rate was significantly lower in the high LAR group compared to the low LAR group (log-rank test p < 0.001). Table 2 shows that in the unadjusted crude model, each unit increase in log (LAR) was associated with a 2.31-fold increase in mortality risk (HR 2.31, 95% CI: 1.61, 3.29, p < 0.001). In the minimally adjusted model (sex, age), the risk increased to 2.32-fold (HR 2.32, 95% CI: 1.62, 3.32, p < 0.001). In the fully adjusted model with additional covariates, the risk increased to 2.51-fold (HR 2.51, 95% CI: 1.73, 3.65, p < 0.001). Sensitivity analysis with LAR as a categorical variable yielded similar results. In the fully adjusted model, the HR and 95% CI for the middle and high LAR tertiles were 1.41 (0.68, 2.91) and 2.52 (1.17, 5.46), respectively (p for trend = 0.015).

**Table 2:** Multivariable Cox proportional hazards regression for effect of LAR.

Exposure	Non-adjusted		Adjust I		Adjust II	
	HR (95%CI)	P-value	HR (95%CI)	P-value	HR (95%CI)	P-value
Log (LAR)	2.31 (1.61, 3.29)	<0.001	2.32 (1.62, 3.32)	<0.001	2.51 (1.73, 3.65)	<0.001
LAR tertile						
Low	Reference	–	Reference	–	Reference	–
Middle	1.47 (0.73, 2.98)	0.283	1.47 (0.73, 2.98)	0.284	1.41 (0.68, 2.91)	0.355
High	2.08 (1.07, 4.04)	0.032	2.09 (1.07, 4.07)	0.031	2.30 (1.15, 4.59)	0.018
P for trend		0.029		0.028		0.015

The non-adjusted model did not adjust for any variables. Adjust I model adjusted for age and sex. Adjust II model adjusted for CHOL, BIL-INDIR, PaCO_2_, PaO_2_.

### 3.3 Linear relationship between log (LAR) and 28-day ICU mortality subgroup analyses

**Fig 3:**
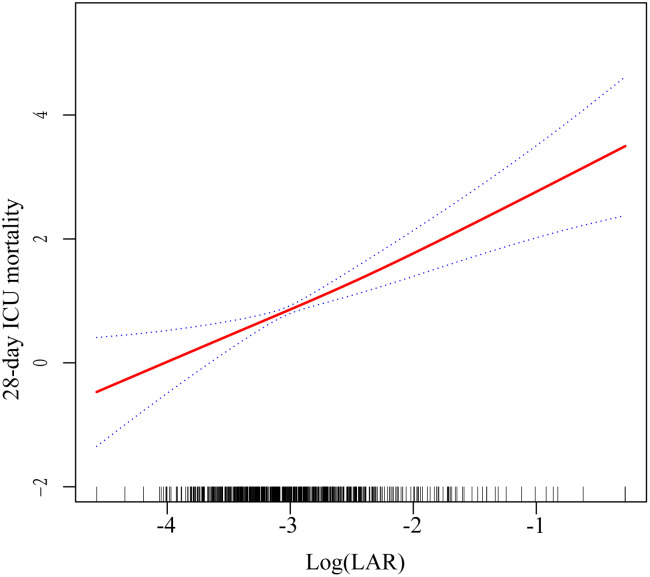
Hazard ratios (95% confidence intervals) for the association between log-transformed lactate-to-albumin ratio (Log_LAR) and 28-day ICU mortality in paediatric severe pneumonia patients. The red line represents the estimated risk of 28-day ICU mortality, while the blue lines represent the 95% confidence intervals after adjustment for covariates.

To further explore the relationship between log (LAR) and 28-day ICU mortality, restricted cubic splines (RCS) were used for fitting (Fig 3). The results revealed a significant linear relationship between log (LAR) and 28-day ICU mortality. As log (LAR) increased, 28-day ICU mortality showed a linear upward trend. Specifically, when log (LAR) was low (e.g., between −4 and −3), the risk of 28-day ICU mortality was relatively low; as log (LAR) increased (e.g., from −3 to −1), the risk of 28-day ICU mortality significantly increased. The RCS fitting demonstrated that this linear relationship was stable across the entire range.

### 3.4 Subgroup analysis

**Fig 4:**
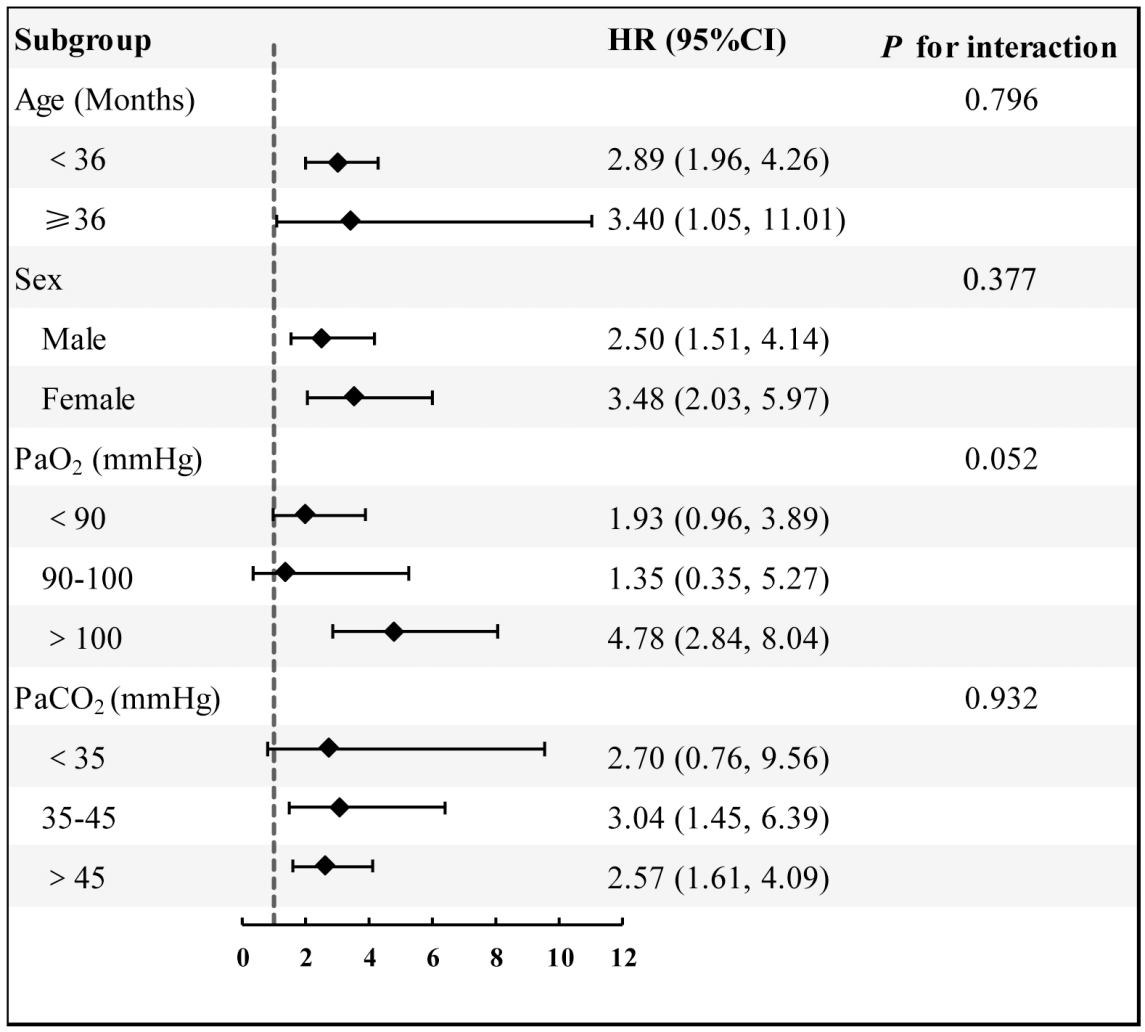
Subgroup analysis of the association between log-transformed lactate-to-albumin ratio (Log_LAR) and 28-day ICU mortality in paediatric severe pneumonia patients after adjustment for covariates.

To further assess the stability of the association between log (LAR) and 28-day mortality, subgroup analyses were conducted (Fig 4). Subgroups were stratified by age (<36 months vs. ≥ 36 months), sex, partial pressure of oxygen (PaO_2_: < 90 mmHg, 90–100 mmHg, > 100 mmHg), and partial pressure of carbon dioxide (PaCO_2_: < 35 mmHg, 35–45 mmHg, > 45 mmHg). Interaction analysis showed that the association between log (LAR) and 28-day mortality remained consistent across subgroups and was not significantly influenced by age, sex, PaO_2_, or PaCO_2_ levels (all interaction p-values >0.05). These results indicate that the association between log (LAR) and 28-day mortality is highly stable and applicable to pediatric severe pneumonia patients across different clinical backgrounds.

## 4 Discussion

This single-center retrospective study found that LAR has good predictive ability for 28-day ICU mortality in pediatric severe pneumonia patients. The results showed that higher LAR levels were significantly associated with an increased risk of 28-day ICU mortality, and patients in the highest LAR tertile had a significantly lower probability of survival compared to those in the lowest tertile. The Cox proportional hazards model further validated the robust association between log(LAR) and 28-day ICU mortality. Subgroup analyses showed that this association remained consistent across different age groups, genders, and oxygenation statuses. These findings suggest that LAR, as a comprehensive biomarker reflecting metabolic and inflammatory states, can be used for early risk stratification in pediatric severe pneumonia patients.

Our study revealed a significant linear relationship between LAR and 28-day ICU mortality. As log (LAR) increased, the risk of death showed a linear upward trend. This result is consistent with the findings of Wang et al., who reported that LAR was an independent risk factor for in-hospital mortality in pediatric ICU patients, with its predictive value surpassing that of lactate alone [15]. Our study further validates the prognostic value of LAR in pediatric severe pneumonia patients, particularly in the high LAR group, where cumulative survival rates were significantly lower than in the low LAR group. This suggests that LAR not only reflects metabolic dysfunction and inflammatory status but also effectively predicts short-term prognosis. This finding provides a theoretical basis for early intervention in pediatric severe pneumonia patients, especially in resource-limited settings, where LAR can help clinicians allocate medical resources more effectively and prioritize high-risk patients.

Recent studies further support the prognostic value of LAR and serum albumin in critically ill pediatric patients. In children with nosocomial infections, the lactate/albumin ratio measured at 24 hours after admission showed the highest predictive value for mortality, indicating its potential role in dynamic risk assessment [16]. In addition, a multi-center analysis revealed that hypoalbuminemia at PICU admission was independently associated with increased mortality, longer ICU stays, and higher demand for organ support therapies, underscoring the critical role of albumin in pediatric critical illness [17]. These findings further validate the clinical utility of LAR as a simple, cost-effective biomarker for early prognostic evaluation.

From a physiological perspective, elevated LAR reflects two critical pathophysiological processes: increased lactate levels and decreased albumin levels. Lactate is a marker of tissue hypoxia and metabolic dysfunction, and its elevation typically indicates inadequate tissue perfusion and a shift from aerobic to anaerobic metabolism [18,19]. In severe pneumonia patients, pulmonary inflammation and infection can impair oxygenation, leading to systemic tissue hypoxia and increased lactate production [20,21]. Additionally, inflammatory responses and infections activate the immune system, releasing large amounts of inflammatory mediators that exacerbate tissue hypoxia and lactate accumulation [22,23]. Moreover, recent evidence suggests that elevated LAR may also serve as a biomarker of microcirculatory injury, reflecting impaired microvascular density and flow in critically ill patients with sepsis [10].

On the other hand, decreased albumin levels reflect nutritional status and the severity of inflammation [6,24,25]. Albumin, the primary plasma protein, plays roles in maintaining colloid osmotic pressure, regulating inflammation, and exerting antioxidant effects [26]. In severe pneumonia patients, inflammation and infection reduce albumin synthesis, increase its breakdown, and enhance vascular permeability, leading to hypoalbuminemia [27]. Hypoalbuminemia exacerbates tissue edema, impairs immune function and drug metabolism, and worsens the patient’s condition [28,29].

Our study also found that LAR was associated with multiple clinical indicators, including white blood cell count, red blood cell count, platelet count, and partial pressure of oxygen. These abnormal changes further support the rationale for LAR as a comprehensive prognostic marker. For example, patients in the high LAR group typically had higher white blood cell counts and lower partial pressure of oxygen, suggesting more severe inflammation and tissue hypoxia. These findings align with those of Lichtenauer et al., who noted that LAR comprehensively reflects inflammatory and metabolic dysfunction, making it a valuable prognostic tool in critically ill patients [7].

Additionally, our study validated the stability of the association between LAR and 28-day mortality through subgroup analysis. The results showed that this association was not influenced by age, sex, partial pressure of oxygen, or partial pressure of carbon dioxide levels. This finding further supports LAR as a reliable prognostic marker applicable to pediatric severe pneumonia patients across different clinical contexts. Similar results were reported by Le et al., who found that LAR achieved an AUC of 0.91 in pediatric septic shock patients, demonstrating excellent prognostic predictive ability [13].

However, this study has several limitations. First, although the dataset is well-curated, it is derived from a single institution in China, which may limit the generalizability of the findings to other geographic regions, healthcare systems, or patient populations. Differences in clinical practices, patient demographics, and resource availability may affect the applicability of our results in broader settings. Second, the study’s reliance on existing medical records for data collection might result in incomplete or inconsistent data, potentially impacting the robustness of the analyses. Finally, the retrospective observational design precludes causal inference. Future studies should include larger, prospectively collected, multicenter cohorts to externally validate the prognostic value of LAR and enhance the generalizability of these findings.

## 5 Conclusions

This study is the first to demonstrate an independent association between the lactate-to-albumin ratio (LAR) and increased 28-day ICU mortality risk in pediatric patients with severe pneumonia. Higher LAR levels were consistently linked to worse outcomes across multiple patient subgroups. These findings suggest that LAR may serve as a valuable prognostic biomarker to support early risk stratification and guide clinical decision-making in critically ill pediatric pneumonia patients.

## Supporting information

S1 Table Baseline characteristics of pediatric pneumonia patients admitted to the ICU by survival status. (DOCX)
