# Supplementary material for: Association between lactate-to-albumin ratio and 28-day ICU mortality in pediatric severe pneumonia patients
Source: PLoS One. 2025 Sep 12;20(9):e0331486. doi: 10.1371/journal.pone.0331486 (PMC12431254; doi:10.1371/journal.pone.0331486)
Supplement: S1 Table — (DOCX) [file pone.0331486.s001.docx]

**Table S1:** Baseline characteristics of pediatric pneumonia patients admitted to the ICU by survival status.

| **28-day ICU mortality** | **Survival** | **Non-survival** | **P-value** |
| --- | --- | --- | --- |
| N | 559 (90.60%) | 58 (9.40%) | - |
| LAR | 0.05 (0.04-0.07) | 0.06 (0.04-0.11) | 0.005 |
| Age (months) | 4.68 (1.68-19.08) | 6.06 (2.79-10.80) | 0.496 |
| Sex (Male) | 331 (59.21%) | 33 (56.90%) | 0.733 |
| WBC (×10^9/L) | 10.38 (6.92-14.42) | 11.91 (7.28-16.85) | 0.181 |
| RBC (×10^12/L) | 3.80 (3.27-4.28) | 3.64 (3.29-4.13) | 0.515 |
| PLT (×10^9/L) | 295.50 (188.00-400.50) | 289.75 (176.38-391.25) | 0.770 |
| HGB (g/L) | 107.00 (94.50-120.67) | 102.50 (90.12-118.38) | 0.142 |
| ALB (g/L) | 37.10 (33.10-40.55) | 37.85 (31.62-40.65) | 0.835 |
| CHOL (mmol/L) | 3.01 (2.32-3.71) | 2.78 (2.41-3.47) | 0.467 |
| TG (mmol/L) | 0.92 (0.65-1.28) | 1.01 (0.63-1.32) | 0.652 |
| BIL-DIR (μmol/L) | 3.00 (1.60-8.28) | 2.30 (1.70-6.40) | 0.432 |
| BIL-INDIR (μmol/L) | 5.80 (3.40-16.10) | 4.30 (2.80-8.70) | 0.008 |
| Scr (μmol/L) | 41.00 (35.00-51.00) | 42.00 (32.25-55.00) | 0.899 |
| ALT (U/L) | 21.00 (14.00-34.50) | 29.00 (16.17-58.75) | 0.006 |
| AST (U/L) | 45.00 (33.00-75.00) | 53.50 (36.25-110.00) | 0.029 |
| Na^+^ (mmol/L) | 137.00 (134.92-139.00) | 136.71 (135.00-140.15) | 0.757 |
| K^+^(mmol/L) | 4.03 (3.70-4.37) | 4.00 (3.55-4.46) | 0.719 |
| Lactate (mmol/L) | 1.80 (1.30-2.44) | 2.05 (1.38-3.68) | 0.005 |
| PaCO_2_ | 42.40 (36.35-51.40) | 48.12 (38.01-56.70) | 0.022 |
| PaO_2_ | 114.08 (82.25-147.69) | 91.95 (66.05-120.17) | 0.003 |

Continuous variables in the table are expressed as median (interquartile range), while categorical variables are expressed as frequency (%). Abbreviations: LAR, lactate-to-albumin ratio; WBC, white blood cell count; RBC, red blood cell count; PLT, platelet count; HGB, hemoglobin; ALB, albumin; CHOL, cholesterol; TG, triglycerides; BIL-DIR, direct bilirubin; BIL-INDIR, indirect bilirubin; Scr, serum creatinine; ALT, alanine aminotransferase; AST, aspartate aminotransferase; Na^+^, sodium; K^+^, potassium; PaCO_2_, partial pressure of carbon dioxide; and PaO_2_, partial pressure of oxygen.
